# Early Interleukin-6 and Slope of Monocyte Human Leukocyte Antigen-DR: A Powerful Association to Predict the Development of Sepsis after Major Trauma

**DOI:** 10.1371/journal.pone.0033095

**Published:** 2012-03-14

**Authors:** Aurélie Gouel-Chéron, Bernard Allaouchiche, Caroline Guignant, Fanny Davin, Bernard Floccard, Guillaume Monneret, for AzuRea Group

**Affiliations:** 1 Service de réanimation, Hôpital Edouard Herriot - Hospices Civils de Lyon, Lyon, France; 2 Laboratoire d’immunologie cellulaire, Hôpital Édouard Herriot - Hospices Civils de Lyon, Lyon, France; 3 Laboratoire Commun de Recherche HCL-Biomérieux, Hôpital Édouard Herriot - Hospices Civils de Lyon, Lyon, France; 4 EA 4174, Hémostase, Inflammation et Sepsis, Hospices Civils de Lyon - Université Claude Bernard Lyon 1, Lyon, France; University of Ottawa, Canada

## Abstract

**Objective:**

Major trauma is characterized by a pro-inflammatory response, followed by an immunosuppression. Recently, in trauma patients, the lack of recovery of monocyte Human Leukocyte Antigen DR (mHLA-DR, a biomarker of ICU-acquired immunosuppression) between days 1–2 and days 3–4 has been demonstrated to be independently associated with sepsis development. The main objective of this study was to determine whether early measurements of IL-6 (interleukin-6) and IL-10 plasma concentrations (as markers of initial severity) could improve, in association with mHLA-DR recovery, the prediction of sepsis occurrence in severe trauma patients.

**Design:**

Prospective observational study over 24 months in a Trauma ICU at university hospital.

**Patients:**

Trauma patients with an ISS over 25 and age over 18 were included.

**Measurements and Main Results:**

mHLA-DR was assessed by flow cytometry, IL-6 and IL-10 concentrations by ELISA. 100 consecutive severely injured patients were monitored (mean ISS 37±10). 37 patients developed sepsis. IL-6 concentrations and slope of mHLA-DR expression between days 1–2 and days 3–4 were significantly different between septic and non-septic patients. IL-10 was not detectable in most patients. After adjustment for usual clinical confounders, when assessed as a pair, multivariate logistic regression analysis revealed that a slope of mHLA-DR expression (days 3–4/days 1–2)≤1.1 and a IL-6 concentration ≥ 67.1 pg/ml remained highly associated with the development of sepsis (adjusted OR 18.4, 95% CI 4.9; 69.4, p = .00002).

**Conclusions:**

After multivariate regression logistic analysis, when assessed as a pair, a high IL-6 concentration and a persistent mHLA-DR decreased expression were found to be in relation with the development of sepsis with the best predictive value. This study underlines the usefulness of daily monitoring of immune function to identify trauma patients at a high risk of infection.

## Introduction

Severe injuries induce a systemic inflammatory response that may be followed by an anti-inflammatory response [1], which contributes to a state of transient immunosuppression [2], [3], [4]. The latter is believed to be directly responsible for a detrimental outcome in trauma patients and for lowering the resistance to nosocomial infections in patients who have survived initial resuscitation [5], [6], [7]. In the absence of specific clinical signs of immune dysfunction in intensive care patients, biomarkers of immunosuppression are clearly highly desirable. To date, diminished expression of Human Leukocyte Antigen DR on circulating monocytes (mHLA-DR) is widely accepted as a reliable indicator of immunosuppression in Intensive Care Unit (ICU) patients. Recently, a decreased in mHLA-DR expression has been shown in severely injured trauma patients. Most importantly, it appeared that the slope of mHLA-DR recovery was a significant predictor of forthcoming sepsis [8]. Concerning the first inflammatory step of trauma pathophysiology, a large number of biomarkers has been studied, mainly with regard to severity. Among them, Interleukin-6 (IL-6), which concentration is in relation with the severity of injury [9], [10], [11], and IL-10, a major component of the compensatory anti-inflammatory cascade [12], have been demonstrated of interest. To our knowledge, no study has been reported so far on concomitant assessment of those both cytokines and mHLA-DR in severely injured trauma patients. As immunosuppression is hypothesized to be proportional to the intensity of initial tremendous inflammation, we reasoned that cumulative information from both initial cytokine response and delayed evolution of mHLA-DR may provide improved information regarding the risk of secondary infection development.

The main objective was thus to demonstrate in a cohort of severe trauma patients that the combination of an early marker of inflammation and mHLA-DR kinetics is a better predictor of sepsis occurrence than each marker alone.

## Materials and Methods

This work belongs to a global study on ICU-induced immune dysfunctions. It has been approved by our Institutional Review Board for ethics (“Comité de Protection des Personnes”) which waived the need for informed consent because biomarkers expression was measured on residual blood after completing routine follow-up. The study is registered at French Ministry of Research and Teaching (#DC-2008-509) and is also recorded in our commission for informatics and freedom (“Commission Nationale de l’Informatique et des Libertés”). As recommended, patients or their family were orally informed of a samples collection and of the purpose of the study.

This prospective observational study was carried out over a 24-month period (July 2008 to June 2010). Management of trauma by specialized physicians were guided with several protocols, approved and followed by all the medical staff, and two medical meeting took care in the ICU every day to discuss the evolution and the treatment of patients. Inclusion criteria were an Injury Severity Score (ISS) [13], [14] of more than 25, admission to the ICU and length of stay in ICU ≥ 3 days. Clinical exclusion criteria were age of less than 18 years, ISS of less than 25, aspiration pneumonia or gut perforation during the first hours following trauma, chronic corticosteroid therapy, and death in the first 48 hours after admission. Patients admitted on a Saturday were excluded because mHLA-DR cannot be measured on day 1 or 2 (blood samples were not collected on Saturdays or Sundays, when the laboratory did not operate).

All patients admitted were followed up prospectively until day 14 by daily clinical examination and biological tests. During follow-up, clinical and biological data were collected. The data collection comprised demographic characteristics (age and gender), infection characteristics (source, microorganisms identified, delay between trauma, and onset of sepsis), and outcome at 28 days (death or survival, with collection of the cause of death). Therapeutic data were also collected (a) on admission to the trauma room (the need for inotropic or vasoactive support and blood products [packed red blood cells, fresh frozen plasma, platelets concentrates, and albumin] and their quantities used to sustain a mean arterial pressure [MAP] up to 70 mm Hg [or 90 mm Hg in the case of head injury], and the type and quantity of prophylactic antibiotics) and (b) during support (number of ventilator days, quantity and type of vasoactive support and of blood products, and use of massive transfusion, which was defined as more than 10 units of packed red blood cells [15], [16] or the replacement of the patient’s total blood volume [17] over a 24 hour period). Creatinine, lactate concentration, and abnormal biphasic waveform (BPW) (MDAII analyzer, BioMérieux, Marcy L’Etoile, France) [18] were measured daily. Three clinical scores were recorded: ISS on admission (range of 0 to 75), initial severity of disease as assessed by the new Simplified Acute Physiology Score II (SAPS II) (range of 0 to 164) [19] and the Sepsis-related Organ Failure Assessment (SOFA) score (range of 0 to 24) on admission and every day during follow-up [20]. Severe brain and thoracic injuries, defined as an Abbreviated Injury Score (AIS) ≥ 3, which are well established as risk factors for sepsis development, were also taken into account [14].

This discovery cohort differed from 15% from that of our previous study [8], as some patients were added (this study was designed a year later) and other excluded due to absence of frozen collected plasma.

The American College of Chest Physicians/Society of Critical Care Medicine Consensus Conference definition of sepsis was used for this study [21], namely the presence of an identifiable site of infection and evidence of a SIRS on the basis of at least two of the following criteria: (a) body temperature of greater than 38°C or of less than 36°C, (b) heart rate of greater than 90 beats per minute, (c) respiratory rate of greater than 20 breaths per minute or hyperventilation as indicated by an arterial partial pressure of carbon dioxide (PaCO_2_) of less than 32 mm Hg (less than 4.3 kPa), and (d) a white blood cell count of greater than 12,000 cells/mm^3^ or of less than 4,000 cells/mm^3^ or the presence of more than 10% immature neutrophils. The onset of sepsis was defined, as recommended by the Consensus Conference [21], as the day on which the site of infection was identified.

The final diagnosis of sepsis was retrospectively established by two experts assessing the complete medical data, not involved in case management and blind of the results. Diagnoses of pneumonia and urinary infection were established according to the guidelines of the American Thoracic Society and the Infectious Diseases Society of America [22] and of the Centers for Disease Control [23], respectively. Physicians were not informed of mHLA-DR results. BPW was also determined as it may be used as an indicator of sepsis development [24], [25], [26], [27].

Ethylenediaminetetraacetic acid (EDTA)-anticoagulated blood samples were collected at 8 a.m. every 2 days after injury (on Mondays, Wednesdays, and Fridays) (that is, at days 1–2, days 3–4, days 5–6, days 7–8, days 9–10, and days 11–12). Data collected on days 5–6, days 7–8, days 9–10, and days 11–12 are not presented, as occurring after sepsis development. Flow cytometric (FC500; Beckman Coulter, Hialeah, FL, USA) expression of mHLA-DR was assessed on arterial, venous, or capillary blood. Samplings were acquired through central venous or arterial catheter, as the severity of the trauma required such equipments. This avoided bias in the blood collection and platelet activation associated with capillary blood sampling. Blood samples were stored immediately at 4°C and stained within 2 hours after collection, in accordance with the standardization recommendations for mHLA-DR measurement [28], [29]. Staining and cell acquisition were undertaken as described in the European standardized protocol. Monoclonal antibodies and their respective isotype controls were used according to the manufacturers’ recommendations: fluorescein isothiocyanate (FITC)-labeled anti-CD14 (10 µL; Immunotech, Marseille France) and phycoerythrin (PE)-labeled anti-HLA-DR (20 µL; BD Pharmingen, San Diego, CA, USA) per 100 µL of whole blood. Monocytes were characterized on the basis of their CD14 expression. Results were expressed as the number of anti-HLA-DR antibodies per cell (AB/C) (normal>15,000), which is correlated with the number of HLA-DR molecules expressed on each monocyte [28]. Control beads (Flowcheck, BeckmanCoulter) and stabilized blood (Immunotrol, BeckmanCoulter) were used on daily basis to check the flow cytometer performance and to valid the accuracy. In addition, calibration beads (Quantibrite®, Becton Dickinson) were monthly run in order to provide results as AB/C. The remaining plasma collected was separated in aliquot and frozen at -80°C.

As we decided to assess the early cytokine response induced by trauma, the two first samples of each patient were analyzed (i.e. days 1–2 and days 3–4). Plasma levels of IL-6 and IL-10 were measured using commercial ELISA kits, according to the manufacturers’ recommendations (Human IL-10 and IL-6 Duoset, R&D Systems, Inc., Minnesota, United States). Recombinant human IL-6 and IL-10 were used to produce a standard curve. The detection limits were 7.8 pg/ml and 9.4 pg/ml respectively. Every sample was analyzed in duplicate.

As sepsis, by itself, can modify mHLA-DR and interleukins concentration, immunological data were censored and excluded from the analysis once the onset of sepsis was established, thereby precluding calculation of a difference in mHLA-DR expression between septic and non-septic patients at days 7–8, 9–10, and 11–12 (because of insufficient numbers of values for statistical analysis). As a consequence, every patient who had a mHLA-DR expression measured at days 3–4 did not present sepsis.

All data were verified for normality using the Kolmogorov-Smirnov test. Baseline characteristics were described by frequency, median and interquartile range (IQR) or, where appropriate, mean ± standard deviation. Patients were separated into two groups, according to the development or not of sepsis. Groups were compared using the Mann-Whitney *U* test for continuous non-parametric variables, the independent paired *t* test for continuous parametric variables and the chi-square test for categorical data. Immunological values were stratified according to the best threshold chosen using a Receiver operating characteristic (ROC) curves analysis (i.e., maximized sensitivity and specificity). ROC curves and the areas under the curve were calculated for the slope in mHLA-DR between days 1–2 and days 3–4. mHLA-DR expression and IL-6 concentration were assessed as a pair leading to four categories : mHLA-DR and IL-6 below their respective optimal thresholds, one above and one below (constituting two groups) and finally both above thresholds.

Univariate and different multivariate logistic regression analyses were used to identify variables associated with the risk of infection and assessed by odds ratios (OR) and 95% confidence intervals (CI), using the different four groups constituted.

A 5% or lower p-value is considered to be statistically significant. The Bonferroni correction was used to avoid spurious results from the multiple statistical tests performed simultaneously. The alpha value for three tests was .016. Medcalc software, version 9.6.4.0 (MedCalc, Mariakerke, Belgium) was used to perform the statistical analyses.

## Results

One hundred patients met the inclusion criteria between July 2008 and June 2010. Patients’ characteristics, therapeutic data and immunological data are shown in Table 1 and Table 2. During follow-up, no differences in renal function (assessed by plasma creatinine concentration) or in lactate concentration were observed (data not shown). Regarding the type of blood products transfused, and prophylactic antibiotic, there were no differences between groups (data not shown).

**Table 1:** Clinical patients’ characteristics.

Parameters	Overall populationn = 100	Septicn = 37 (37%)	Non-Septicn = 63 (63%)	p value
Age (years)	37±17	35±16	38±17	.34 *
Male,% (n)	73% (n = 73)	81% (n = 30)	68% (n = 43)	.24 ♦
ISS	37±10	39±8	35±10	.06 *
Severe brain injury,% (n)	38% (n = 38)	57% (n = 21)	27% (n = 17)	.006 ♦
Severe thoracic injury,% (n)	74% (n = 74)	62% (n = 23)	81% (n = 51)	.07 ♦
SAPS II	36.4±15.4	42.5±15.8	32.8±14.1	.002 *
Need for vasoactive support in emergency room,% (n)	26% (n = 26)	38% (n = 14)	19% (n = 12)	.07 ♦
Prophylactic antibiotics administrated in emergency room,% (n)	42% (n = 42)	38% (n = 14)	44% (n = 28)	.66 ♦
SOFA score				
D1	4 [2]; [7]	7 [4.25; 10]	3[2]; [5]	<.0001 ‡
D2	4 [2]; [6]	6[3.5; 10]	2.5 [1]; [5]	<.0001 ‡
D3	3 [2]; [6]	7 [3.5; 9]	2 [1]; [3]	<.0001 ‡
D4	3 [1]; [5]	5.5 [2.5; 8]	2 [1]; [3]	<.0001 ‡
D5	2 [1]; [4]	5 [1.5; 7.5]	1 [1]; [3]	<.0001 ‡
D6	1 [1]; [4]	4 [1.5; 7]	1 [1]; [2]	<.0001 ‡
Shock (need for vasoactive drug on D1–2),% (n)	35% (n = 35)	59% (n = 22)	21% (n = 13)	.0002 ♦
Mechanical ventilation (MV),% (n)	66% (n = 66)	89% (n = 33)	52% (n = 33)	.0004 ♦
Duration of MV, days	6 [3]; [12]	10 [6]; [19]	3 [2; 5.25]	<.0001 ‡
Massive transfusion required,% (n)	32% (n = 32)	40% (n = 15)	27% (n = 17)	.24 ♦
Volume of global transfusion, ml	900 [0; 3,050]	1,800 [150; 4,300]	0 [0; 2,700]	.02 ‡
Length of stay in ICU, days	9.5 [6]; [15]	15 [10.75; 24.25]	7 [5; 10.75]	<.0001 ‡
Non-survivors at day 28,% (n)	5% (n = 5)	8% (n = 3)	3% (n = 2)	.06 ♦

Parametric variables are expressed as mean ± standard deviation, and non-parametric variables as median (interquartile range) or frequencies.

*Independent samples t-test.

‡ Mann & Whitney test.

♦ Chi-square test

Abbreviations: ISS, Injury Severity Score; SAPS, Simple Acute Physiology Score; SOFA, Sequential Organ Failure Assessment; HLA, Human Leukocyte Antigen; ICU, Intensive Care Unit; D, day; IL, Interleukin.

**Table 2:** Immunological patients’ characteristics.

Parameters	Overall population n = 100	Septic n = 37 (37%)	Non-Septic n = 63 (63%)	p value
mHLA-DR levels, antibodies per cell				
D1–2	11,407±5,049	11,120±5,341	11,979±4,451	.48 *
D3–4	12,536±7,508	9,647±5,924	14,120±7,848	.005 *
D5–6	15,429±9,068	11,707±6,004	16,439±9,525	.06 *
Variations in mHLA-DR				
D3–4/D1–2	1.27±.56	.87±.43	1.44±.53	<.0001 *
D5–6/D3–4	1.24±.64	1.32±.82	1.23±.58	.62 *
IL-6 (pg/ml)				
D1–2	63.2 [47.2; 114.9]	95.1 [71.3; 210.3]	55.7 [45.9; 83.8]	.0004 ‡
D3–4	51.1 [44.9; 70.2]	67.2 [45.6; 186]	48 [42.7; 67.3]	.01 ‡
IL-10 (pg/ml)				
D1–2	< 7.8 [< 7.8; 7.8]	< 7.8 [< 7.8; 7.8]	< 7.8 [< 7.8; 7.8]	
D3–4	< 7.8 [< 7.8; 7.8]	< 7.8 [< 7.8; 7.8]	< 7.8 [< 7.8; 7.8]	

Parametric variables are expressed as mean ± standard deviation, and non-parametric variables as median (interquartile range) or frequencies.

* Independent samples t-test.

‡ Mann & Whitney test.

Abbreviations: HLA, Human Leukocyte Antigen; D, day; IL, Interleukin.

Five patients died (three from septic shock and two from cardiogenic shock). No statistical test could be done on mortality considering the poor number of patients.

Thirty-seven patients developed sepsis during follow-up, in a mean delay of 5.1±2.4 days: pneumonia (n = 31) and urinary tract infection (n = 6). Causative bacteria were fairly evenly distributed between Gram-positive (n = 14) and Gram-negative (n = 22) organisms. One patient had a mixed bacterial infection (Gram-positive and -negative).

**Figure 1:**
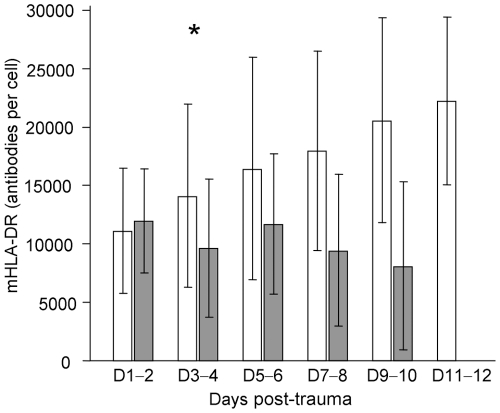
Time course of mHLA-DR expression in trauma patients, with (gray) or without (white bars) sepsis. mHLA-DR is expressed as numbers of anti-HLA-DR antibodies bound per cell (AB/C). Results are expressed as mean ± standard deviation (*t* test, * p<.01). mHLA-DR expression was not different between the two groups at days 1–2, but was significantly lower in septic patients than in non-septic patients at days 3–4.

**Figure 2:**
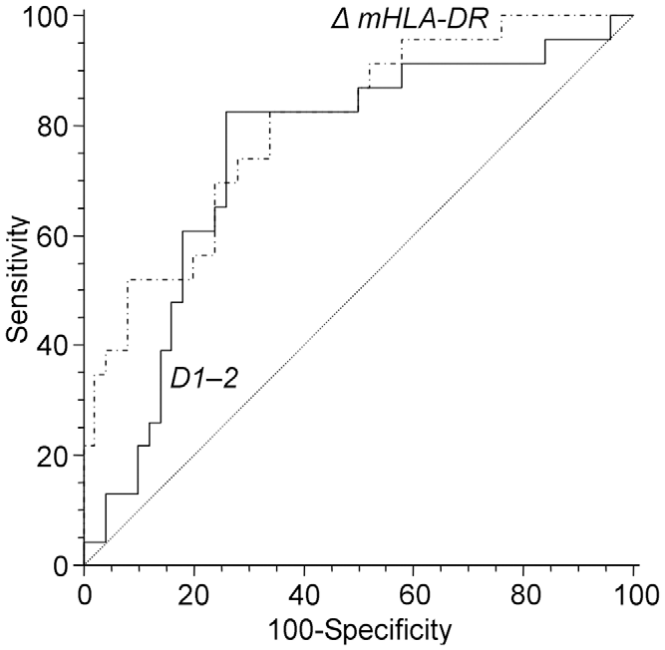
ROC curve of mHLA-DR expression slope (dash line) and IL-6 (full line) for predicting sepsis. Area under curves for mHLA-DR slope (days 3–4 /days 1–2) and IL-6 at days 1–2 were respectively .79 (95%CI .69; .88, p = .0001) and .75 (95%CI .64; .84, p = .0001). The best threshold was 1.1 for mHLA-DR ratio (sensitivity 82.6%, specificity 64.7%) and 67.1 pg/ml for IL-6 concentration (sensitivity 84.6% and specificity 72.5%).

mHLA-DR expression and differences between septic and non-septic patients are shown in Table 2 and Figure 1. As previously described, ratios were calculated between values for two time points. The slope of mHLA-DR expression (days 3–4/days 1–2) showed a highly significant statistical difference between non-septic and septic patients (Table 1). We next established a ROC analysis (Figure 2). Area under curve for mHLA-DR slope (days 3–4 /days 1–2) was .79 (p = .0001, IC95% .69; .88). The best threshold to discriminate septic and non-septic patients (i.e., that maximizes sensitivity and specificity) was 1.1 for mHLA-DR ratio. At that threshold, the test had 82.6% sensitivity, 64.7% specificity, 51% Predictive Positive Value (PPV) and 89% Negative Predictive Value (NPV).

**Figure 3:**
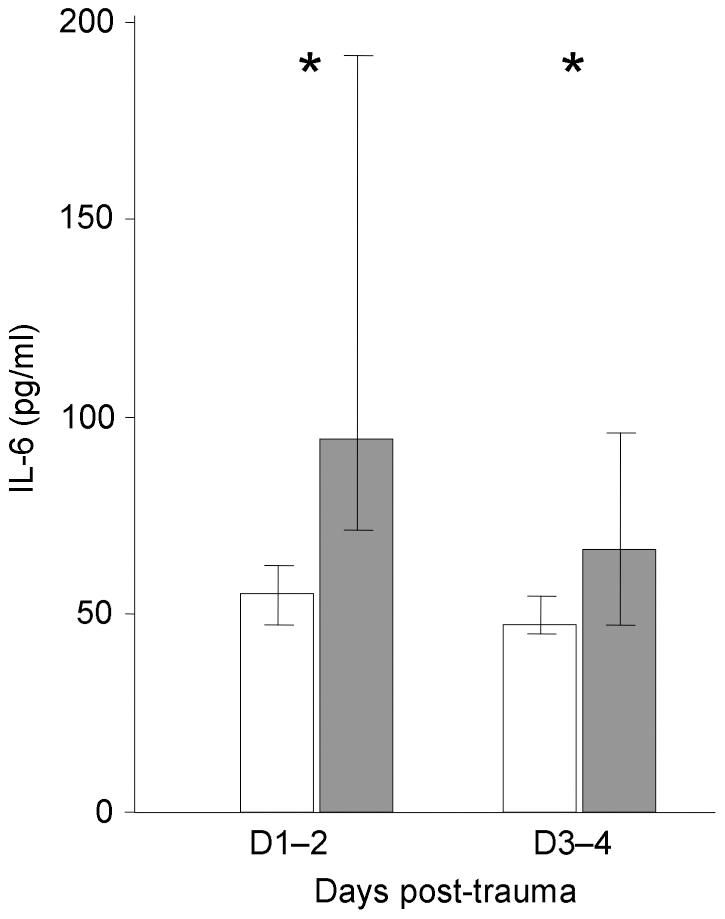
Time course of IL-6 concentration in trauma patients, with (gray) or without (white bars) sepsis. Results (pg/ml) are expressed as median [interquartile range] (Mann & Whitney *U* test, * p<.01). At days 1–2, IL-6 concentration was significantly higher in septic patients than in non-septic patients, as at days 3–4 but with less significance.

At days 1–2, IL-6 concentration was significantly higher in septic patients than in non-septic patients (Table 2, Figure 3). No correlation was found between IL-6 concentration at days 1–2 or 3–4 and mHLA-DR expression (whatever days were considered), severe brain (p = .3) or thoracic injury (p = .06).

The ROC curves analysis, with an area under curve at days 1–2 of .75 (p = .0001, IC95% .64; .84), provided a concentration ≥ 67.1 pg/ml for IL-6 at days 1–2 as the best cut-off values to discriminate septic and non-septic patients. At that threshold, the test had 84.6% sensitivity, 72.5% specificity, 58% PPV and 90% NPV (Figure 2). An IL-6 concentration ≥ 67.1 pg/ml at days 1–2 was highly associated with the development of sepsis and with the need of vasoactive support during the two first days following trauma (*p*<.0001). No correlation was found between mHLA-DR expression (values or slope) and IL-6 concentration, whatever days were considered.

IL-10 was rarely detectable in most trauma patients. Only 16 samples were above detection limit at days 1–2 (11 patients of them developed sepsis, 3 patients died). Among them, 5 samples were still positive at days 3–4, 11 became negative. As IL-10 was present in a few samples, little statistics test could be done. The presence of a detectable IL-10 at days 1–2 was associated with a severe head injury (*p* = .03), mortality at days 28 (*p* = .01), and shock at days 1–2 (*p* = .006), but not with sepsis development (*p* = .3), massive transfusion (*p* = .36) nor major thoracic injury (*p* = .19) (data not shown).

A control group of 18 healthy subjects (age and gender-matched) was selected from among staff members of the intensive care unit and employees working at the laboratory of immunology (data not shown). None of these 18 subjects had detectable IL-6 nor IL-10.

The predictive capacities of clinical and immunological values were tested in a univariate logistic regression analysis. We retained parameters with p<.01. Mechanical ventilation has not been taken into account as it is directly linked to head injury that is included in the statistical model. Finally, only major head injury, SAPS II and immunological values were included in the model (Table 3). As IL-6 concentration and mHLA-DR expression were not correlated, we could analyze them as a pair. Slope of mHLA-DR expression between days 3–4 and days 1–2 and IL-6 concentration at days 1–2 were tested either independently (i.e., tested one by one with clinical variables) or in association as pair (both above or under their best threshold) (Table 4). When analyzed separately in the multivariate analysis, the two immunological data provide an adjusted OR at 9.0 (IC95% 2.5; 32.8, p = .0009) or 10.9 (IC95% 3.0; 39.4, p = .0002).

**Table 3:** Univariate logistic regression analysis according to the biologic test used to predict sepsis development.

		Univariate (n = 100)		
		OR	95%CI	*p* value
Severe head injury	Positive	3.5	1.5 ; 8.4	.004
Severe thoracic injury	Positive	.4	.1 ; .96	.04
Massive transfusion	Positive	1.8	.8 ; 4.4	.16
ISS	≥40	2.5	1.1 ; 5.9	.04
SAPS II	≥37	3.1	1.3 ; 7.1	.009
D3–4/D1–2 mHLA-DR	≤1.1	8.7	2.6 ; 29.5	.0005
D1–2 IL-6	≥67.1	12.0	3.6 ; 40.7	.00006
D3–4/D1–2 mHLA-DR≤1.1 & D1–2 IL-6≥67.1	Positive	21.0	5.8 ; 75.7	.000003
D3–4/D1–2 mHLA-DR>1.1 & D1–2 IL-6≥67.1	Positive	.6	.1 ; 2.5	.49
D3–4/D1–2 mHLA-DR≤1.1 & D1–2 IL-6<67.1	Positive	.4	.1 ; 1.7	.24
D3–4/D1–2 mHLA-DR>1.1 & D1–2 IL-6<67.1	Positive	.05	.007 ; .4	.006

CI, confidence interval; D, days; IL, Interleukin; ISS, Injury Severity Score; mHLA-DR, monocyte human leukocyte antigen-DR; OR, odds ratio; SAPS II, Simple Acute Physiology Score II.

**Table 4:** Different multivariate logistic regression analysis according to the biologic test used to predict sepsis development.

		Multivariate (n = 77)		
		OR	95%CI	*p* value
Severe head injury	Positive	3.3	1.0 ; 11.0	.05
SAPS II	≥37	2.6	.8 ; 8.4	.1
D3–4/D1–2 mHLA-DR	≤1.1	9.0	2.5 ; 32.8	.0009
Severe head injury	Positive	3.5	1.1 ; 11.6	.04
SAPS II	≥37	2.7	.8 ; 8.6	.1
D1–2 IL-6	≥67.1	10.9	3.0 ; 39.4	.0002
Severe head injury	Positive	2.7	.7 ; 9.9	.14
SAPS II	≥37	2.0	.5 ; 7.3	.3
D3–4/D1–2 mHLA-DR≤1.1 & D1–2 IL-6≥67.1	Positive	18.4	4.9 ; 69.3	.00002
Severe head injury	Positive	3.6	1.1 ; 12.1	.03
SAPS II	≥37	2.7	.8 ; 8.7	.09
D3–4/D1–2 mHLA-DR>1.1 & D1–2 IL-6<67.1	Positive	.05	.006 ; .4	.006

CI, confidence interval; D, days; IL, Interleukin; ISS, Injury Severity Score; mHLA-DR, monocyte human leukocyte antigen-DR; OR, odds ratio; SAPS II, Simple Acute Physiology Score II.

Interestingly, when slope of mHLA-DR expression at days 3–4 and IL-6 concentration at days 1–2 were expressed as a pair (both above their respective cut-off), after adjustment for other confounding factors, they appeared as the best predictor for developing sepsis, leading to an elevated adjusted OR at 18.4 (95% CI 4.9; 69.3, p = .00002) (Table 4). The characteristics of the different immunologic statistical tests are presented in Table 5. This reinforces the idea of using a panel of biomarkers to accurately monitor trauma patients.

**Table 5:** Sensitivity, specificity, positive predictive value (PPV), negative predictive value (NPV) for D3–4/D1–2 mHLA-DR, D1–2 IL-6 concentration, and the combination of D3–4/D1–2 mHLA-DR and D1–2 IL-6 concentration, for the diagnosis of sepsis during the intensive care unit (ICU) stay.

	Sensibility	Specificity	PPV	PNV
D3–4/D1–2 mHLA-DR≤1.1	82.6%	64.7%	51%	89%
D1–2 IL-6≥67.1	84.6%	72.5%	58%	90%
D3–4/D1–2 mHLA-DR≤1.1 & D1–2 IL-6≥67.1	69.6%	90.2%	76.2%	86.8%

## Discussion

After initial resuscitation, the course of trauma patients is characterized by an elevated risk of secondary sepsis responsible for prolonged length of stay in ICU, extended duration under mechanical ventilation and increasing morbidity. While recommendations and guidelines are often provided regarding the initial resuscitation, the second step of the disease has remained somehow underinvestigated although it may offers new strategies of prophylactic treatment against infection in the future [30]. Indeed, severe injury is now well known to be followed by a relative immunosuppression with the alteration of a large number of immune functions [31], [32], [33], [34], [35], [36], which can become harmful if it persists. These changes may increase susceptibility to infection.

There are few markers of immune function in ICU. Of them, in vitro LPS-induced TNF-α production and mHLA-DR expression are the most frequently reported. However, mHLA-DR, being a easily measurable surrogate marker of functional testing (comparing to in vitro LPS-induced TNF-α production), has been widely assessed in many ICU conditions [37]. To date, diminished mHLA-DR expression has become the most reliable marker of ICU-acquired immunosuppression [38]. Indeed, every stress situation motivating the admission in ICU (i.e. surgical interventions, sepsis, burns, stroke, trauma or pancreatitis) is characterized by a state of immunosuppression, illustrated by an alteration of mHLA-DR expression. While the potential of mHLA-DR expression to predict outcome remains dependent of sampling time (early measurements are not predictive in all conditions [39], [40], [41], [42], [43]), it has been consensually demonstrated to be a predictor of septic complications in various stress situations [7], [39], [44], [45], [46]. In line, in a previous study, we reported that the pattern of progression of mHLA-DR expression, i.e. the recovery of mHLA-DR expression after days 1–2, was of interest in predicting the development of sepsis after major trauma [8].

IL-6 is a pleiotropic cytokine, undetectable in healthy people, released by a large variety of cellular type [9]. In burned patients or undergoing a major surgery, an early elevation has been shown, from the first to the 6^th^ hour. Its concentration was correlated with development of septic complications or death [47], [48], [49], [50]. In trauma patients, the early elevation of IL-6 has been shown to be related to the severity of trauma, the magnitude of the pro-inflammatory response and complications (septic, multiple organ failure, death) [50], [51], [52], [53], even if no correlation has been found in some other studies [54]. IL-10 is a major anti-inflammatory mediator, undetectable in healthy people. It is elevated in septic patients with a relation between its concentration, the severity of sepsis and death [53], [55], [56]. The ratio IL-6/IL-10 concentration has long been proposed to monitor the immunologic system of the ICU-patient, as it reflects the pro and anti-inflammatory balance (SIRS and CARS) [1], [57]. A decrease of this ratio has been linked to patients’ outcomes and development of sepsis after major surgery [58], [59]. In trauma patients, it seemed that there is a relation between the level of IL-10 expression, the development of sepsis and death [60]. To date and to the best of our knowledge, no study has been reported so far where the concomitant assessment of both cytokines together with mHLA-DR are proven to be useful in the management of severely injured trauma patients.

The descriptive data showed that severe thoracic injury was more frequent in the non septic group. As revealed by univariate logistic regression analysis, thoracic injury seemed protected from sepsis (OR: 0.39). When data were analyzed individually, it appears that patients who had isolated thoracic injury were more often under non-invasive ventilation, when compared to those who had a brain injury associated, who were more often under invasive ventilation and who developed more infections.

In the present study, IL-10 was rarely above detection limit. A relation between a detectable IL-10 and severe head injury has been shown, in accordance with pathophysiological links between brain injury, immunosuppression, and pneumonia development. These results deserve to be further investigated in a severely brain injured patients with ultra sensitive kits for IL-10 measurements. In contrast, IL-6 levels were found to be elevated, with a median concentration at days 1–2 of 63.2 pg/ml [47.2; 114.9], although it was found below concentrations reported in several other works [50], [54], [61]. As previously described [8], [42], we observed a moderate decrease in mHLA-DR expression. Collectively (moderate IL-6 elevation and no detectable IL-10), these data suggest a moderate activation of the inflammatory cascade followed by a mild immunosuppression state induced by trauma. This may be related to the improvement of patients’ resuscitation according to the guidelines. This might also explain the fewer incidences of sepsis and death observed when compared to other epidemiologic studies [62], [63].

Despite this moderate state of immunosuppression (low IL-6 concentration, relatively high mHLA-DR expression comparing to shock septic patients and no detectable IL-10), we still observed a difference between septic and non-septic patients regarding IL-6 concentration and mHLA-DR expression (values and slope of expression). We may thus hypothesize that, after severe trauma, patients who do not restore their mHLA-DR expression and subsequently develop sepsis are those who presented the highest IL-6 values at days 1–2. This association is not a proof of causative relationship and deserves to be further investigated. However, it appears that patients who developed infections are those who were the furthest from the immunologic homeostasis.

As previously described [8], the predictive test (considering sensibility and specificity) of the slope of mHLA-DR expression provided good results, as confirmed by the multivariate analysis (adjusted OR 9.0, 95%CI 2.5; 32.8, p = .0009). Similarly, early IL-6 results were good independent predictors of sepsis occurrence (adjusted OR 10.9, 95%CI 3.0; 39.4, p = .0002).

Most importantly, the major result of this study is that after adjustment for usual clinical confounders (i.e. the presence of a severe head injury and an elevated SAPS II), the combinative test of adding the slope of mHLA-DR expression between days 3–4 and days 1–2 and early IL-6 concentration at days 1–2 was found to be independently associated with the risk of developing sepsis with a significant elevated OR (adjusted OR 18.4, 95%CI 4.9; 69.3, p = .00002). When comparing the statistical characteristics of the tests (Table 5), even if sensibility decreased, the specificity and the PPV sharply increased when combining both markers thus creating a better screening test. Moreover, in multivariate analysis, this association was more predictive than each biomarker by itself, doubling the adjusted OR. As the mean delay of sepsis was 5.1±2.4 days and as the data were censored from the statistical analysis after sepsis occurrence, we assumed that this created a predictive test for sepsis in trauma patients.

In the present study, we reasoned that cumulative information from both initial cytokine response and delayed evolution of mHLA-DR may provide improved information regarding the risk of secondary infection development. Indeed, the association of these two markers partially reflects the immunologic balance. The early hyper-inflammatory response following trauma is characterized by a massive pro-inflammatory mediator release, such as IL-6, which concomitantly induces the production of major anti-inflammatory mediators such as IL-10 and down regulation of mHLA-DR. This reflects a protective mechanism in order to prevent tissue damage induced by uncontrolled inflammation. Thus, as SIRS and CARS are connected, it is likely that the magnitude of the first one is related to the magnitude of the second one [33]. In line, it has been shown in previous studies that the more far the patients were from the immunologic homeostasis (activation or depression), the more they developed infections [64]. This may explain the relevance of the present results.

There are some limitations that need to be acknowledged and addressed regarding the present study. The first limitation is that this study is monocentric and only includes one hundred patients. However it is important to note that such number of inclusions remains high when compared to previous studies. Moreover our cohort was homogenous regarding severity. Even if IL-6 concentration is quite low when compared to previous studies, variations between groups were still found statistically significant. In a previous study in which severe immunosuppression was assessed by low mHLA-DR expression (around 30%), IL-10 measured with an automatic method was also barely detectable [44]. Another limitation concerns the sequence sampling (i.e. every 2 days in the study design). Considering the mean delay of sepsis (5.1 days), one next challenge is to evaluate whether a shortened monitoring in the first 2 days could provide similar predictive information regarding the risk of sepsis. If so, it would increase the time window to develop strategies to control secondary sepsis. Lastly, one limitation refers to the subgroup of patients with head injury. As previously discussed, it is well known that they are at higher risk of sepsis, as shown in Table 1. One would assume the fact that this could represent a bias in our analysis. Nevertheless, by conducting a multivariate analysis, the results were adjusted with confounding parameters, including head injury.

In conclusion, to the best of our knowledge, this is the first study to provide such a high OR to predict the risk of forthcoming sepsis on the basis of biomarkers in trauma patients. Our results highlight the interest to concomitantly assess early value of IL-6 concentration (as a marker for severity) and daily variation of mHLA-DR expression (as a marker for persisting immunosuppression). Thus, a panel of markers could be an innovative approach to follow rapid changes in immune functions after injury. It would permit to identify patients at a high risk of infection. To “pre-empt” the development of sepsis, these patients could receive prophylactic treatments, such as antibiotics [65], immunostimulant therapies using interferon-gamma [66] or granulocyte-macrophage colony-stimulating factor, as proposed in septic shock [31].

### Conclusions

In conclusion, trauma induces a temporary, relative immunosuppression. In this preliminary study, the association between a lack of mHLA-DR recovery and IL-6 concentration at days 1–2 can result in a performance test to predict sepsis. Multicentre studies will have to be designed in order to confirm these promising results in an independent population. Studies should also focus on patients with major head injury and the pathophysiological links with pneumonia development. This study confirms the importance of inflammatory and immunologic monitoring in trauma patients and the pivotal role of immune dysfunction in the increased risk of infection in trauma patients.
